# Full space device optimization for solar cells

**DOI:** 10.1038/s41598-017-12158-0

**Published:** 2017-09-20

**Authors:** Ahmer A. B. Baloch, Shahzada P. Aly, Mohammad I. Hossain, Fedwa El-Mellouhi, Nouar Tabet, Fahhad H. Alharbi

**Affiliations:** 1 College of Science & Engineering (CSE), Hamad Bin Khalifa University, Doha, Qatar; 2 Qatar Environment and Energy Research Institute (QEERI), Hamad Bin Khalifa University, Doha, Qatar

## Abstract

Advances in computational materials have paved a way to design efficient solar cells by identifying the optimal properties of the device layers. Conventionally, the device optimization has been governed by single or double descriptors for an individual layer; mostly the absorbing layer. However, the performance of the device depends collectively on all the properties of the material and the geometry of each layer in the cell. To address this issue of multi-property optimization and to avoid the paradigm of reoccurring materials in the solar cell field, a full space material-independent optimization approach is developed and presented in this paper. The method is employed to obtain an optimized material data set for maximum efficiency and for targeted functionality for each layer. To ensure the robustness of the method, two cases are studied; namely perovskite solar cells device optimization and cadmium-free CIGS solar cell. The implementation determines the desirable optoelectronic properties of transport mediums and contacts that can maximize the efficiency for both cases. The resulted data sets of material properties can be matched with those in materials databases or by further microscopic material design. Moreover, the presented multi-property optimization framework can be extended to design any solid-state device.

## Introduction

Over the past decade, the field of photovoltaics (PV) has advanced extraordinarily in many fronts. Many of the stagnated PV technologies were significantly improved. For example, cadmium telluride (CdTe) solar cell efficiency was increased to 22.1%^1^ after being pinned around 16.5% for 20 years between 1992 and 2012^2,3^. This also happened for Copper indium gallium selenide (CIGS) solar cells; after 15 years of stagnation of the efficiency around 19%^4,5^, the efficiency was improved in the past three years and reached the record of 22.3%^6^. Furthermore, a new family of hybrid perovskite solar cells has emerged in 2012 and has been developing exceptionally since then; its efficiency reached 22.1% in just four years^1^. As for multijunction cells, Fraunhofer Institute for Solar Energy Systems achieved 46.0% efficiency using four-junction cell^1^. Actually, the list of recent interesting developments in the field is huge; thus, we refer the reader to latest comprehensive reviews^7–10^. Basically, there are many reasons for such remarkable developments. The main one–as usual–is economical due to the increased prices and the depletion rates of other fuel sources^11–13^. Scientifically, nanotechnology and materials sciences have been growing exponentially since 1990s^14,15^. This–in turn–enriches PV field which relies heavily on the advances in material sciences. Historically, the first practical realization of solar cell was in the 1950s^16–18^. It was mainly based on crystalline silicon. The space of materials used in solar cells increased considerably in the 1970s as tens of absorbers were considered. The most prominent output of that era were CdTe and CIGS^13,18^. The set of explored absorbers has been expanding since then.

**Figure 1:**
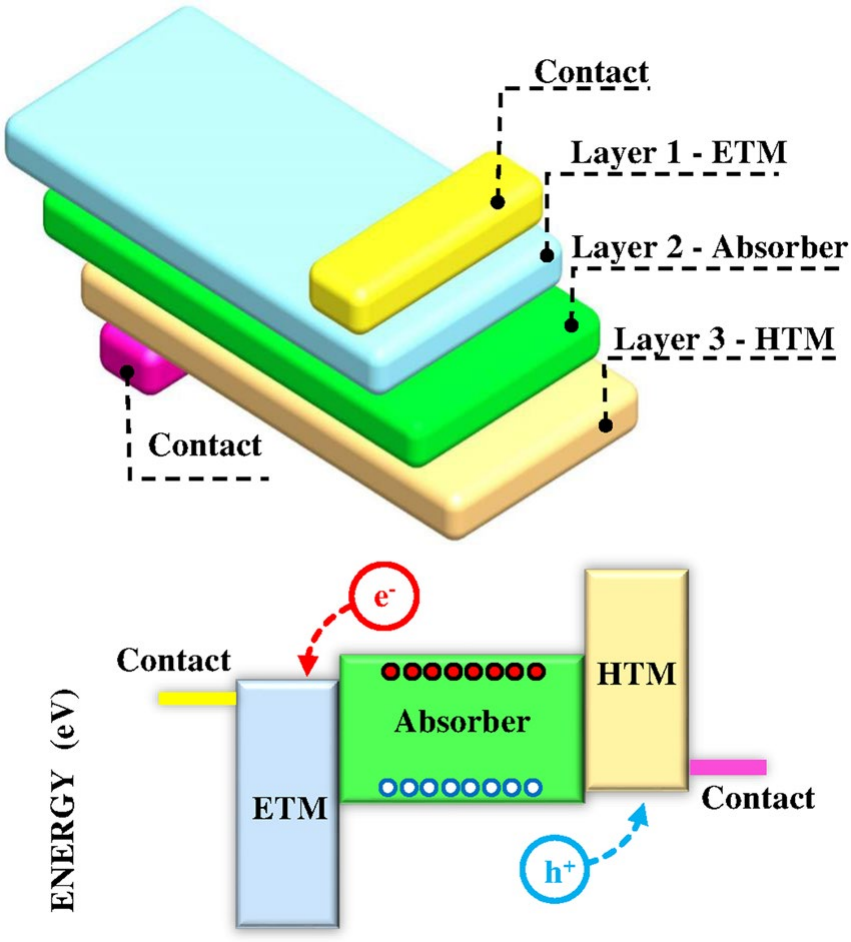
The general device structure of solar cells.

The general structure of solar cells is shown in Fig. 1. Besides the absorber, there are the two contacts and the electron and hole transport materials (ETM and HTM,respectively). More layers could be used for various purposes. In principle, each absorber shall have a unique set of optimally matching materials to maximize the cell efficiency. However, a reduced number of materials is used in different solar cell technologies. For example, cadmium sulfide (CdS) is commonly used for CdTe^19^, CIGS^20^, Cu_*x*_S^21^, and InP^22^ cells as ETM. Also, TiO_2_ is used as ETM in a very wide set of solar cells technologies^13,23^. There are many other examples as well for the use of a particular “non absorbing” material in multiple solar cell technologies. Such coincidences cannot be generally attributed to a device optimization process; but, paradigms and experiences play a major role –at least– at the first stage of development. Furthermore, it is practically very arduous to experimentally identify the best matching device materials for a given absorber. Thus, it is essential to rely on computational device design and optimization.

Solar cell design and optimization needs multi-scale computational approaches. On one hand, materials’ properties are determined by their atomic and micro structures; on the other hand, the device operation can be effectively described at the macroscopic level by solving the equations that govern the light absorption and charge of dynamics across the multi-layered device. Despite the wealth of experimental data available, it is not comparable to the space of materials that can be explored computationally. Most of the solar-cell related microscopic computational efforts were directed towards the device design, analysis and the calculation of the optoelectronic properties of the absorbing material. However, few general scope multi-scale computational efforts^24–28^ were proposed recently and the field is gaining more attention.

Here, we introduce and deploy a full space material-independent optimization to improve the design of solar cells by identifying a material data set for maximum conversion efficiency and for targeted functionality for each layer. A large set of parameters shall be adjusted concurrently to maximize the efficiency of the cell. The range of each parameter is only restricted by the essential physical constraints to ensure full-space optimization. In the proposed approach, the coupled set of equations are solved simultaneously for the objective function and variables. The cell design is optimized by identifying the properties of the optimal matching materials for a given absorber and a set of values of various physical parameters is determined for all the other layers. There are many reported works in this regard^29–35^. However, they don’t span the full possible space. In general, they can be classified into two groups. In the first one, the materials making the different layers such as the absorber, ETM and HTM are well defined. The optimization process, determines the materials characteristics that can be modified experimentally such as the thickness, the doping level and carrier mobilities^29–32^. In the second group, the materials are not predefined. Instead,the key material characteristics that impact the cell performance such as energy gap and electron affinity, are varied within a defined range until the optimum value of the cell performance is obtained. For example, Minemoto and Murata try to adjust the band offsets in perovskite solar cells to maximize the efficiency^33^. For FeS_2_, Altermatt *et al*.^34^ studied optimizing different device aspects by carrying out parametric analysis of single variables such as diffusion length on the performance of solar cells.

In this work, the optimization is carried out using optimization toolboxes in MATLAB^36^ which is interfaced to the one-dimensional (1D) Solar Cell Capacitance Simulator (SCAPS) for device simulation^37,38^. The proposed scheme identifies the needed materials’ properties for maximum conversion efficiency. Two important cases in the field; namely PSC and CIGS are considered, where the approach is implemented to determine the practical efficiency limit of CH_3_NH_3_PbI_3_ solar cells and to identify some possible ETMs for CIGS cell that are free of toxic elements. For PSC under AM1.5g spectrum, it is found that an efficiency of 26.6% can be achieved if the crystal quality of used materials is optimum and; this is reduced to 23.4% if the minimally reported deep defect level is considered. Numerically, both the local and global minima solvers resulted in almost the same efficiency with standard deviations of 7.35 × 10^−3^ and 7.5 × 10^−4^ for both cases but with different optimum data sets. It was found that in term of the required computational cost and maximized objective function, gradient based optimization methods perform better than global algorithms. This is due to the smoothness of physical models and convex nature of the objective function. The second implementation related to cadmium-free CIGS solar cell results in a maximum efficiency of 22.04%. The needed properties of electron-transport medium are identified. Furthermore, the approach can be applied to optimize the complete device structure of any solar cell and identify the properties of the optimal matching materials. Actually, it can be extended to any solid-state device design whenever full space optimization is required.

## Full-Space Device Design Optimization

**Figure 2:**
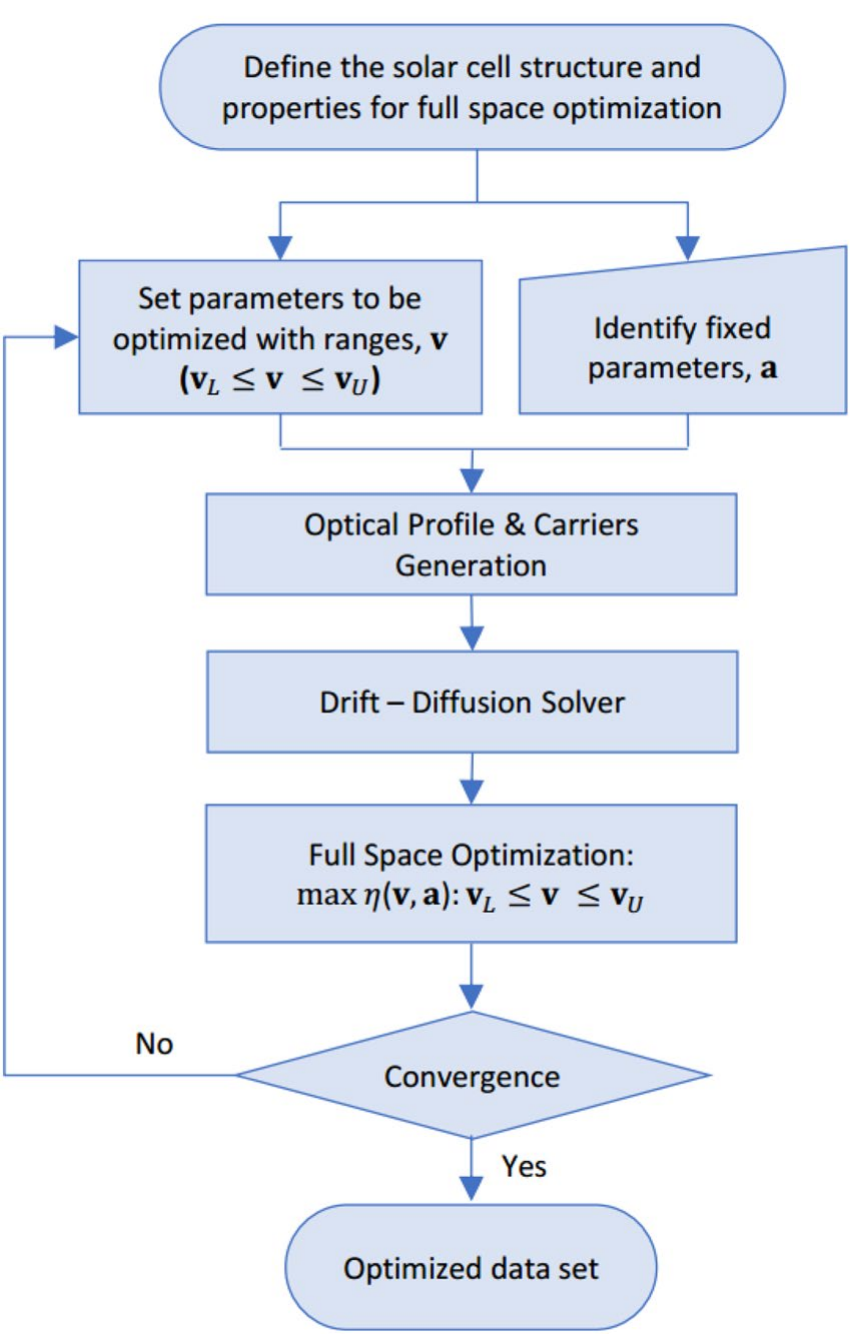
The used scheme for full space device design optimization.

The adopted scheme for full-space device design optimization is sequential as shown in Fig. 2. It is composed of two parts. The first one is the device simulation module, where there are many convenient tools^38^; as aforementioned, in this work, SCAPS is used. Giving two input vectors ${\bf{v}}\in {{\mathbb{R}}}^{N}$ (combing all *N* parameters to be optimized) and ${\bf{a}}\in {{\mathbb{R}}}^{M}$ (combing all other needed *M* fixed parameters), it solves the optically excited charge generation, 1D Poisson’s equation, transport equation, and continuity equations and calculates the efficiency (*η*(**v**, **a**)) of the cell based on the inputs. For higher dimensionality, other tools can be used if needed. **v** and **a** shall cover all the required input parameters to run the simulation. The second module is the numerical optimization tool. It maximizes the objective function *η*(**v**, **a**) by varying **v** based on some physical constraints which themselves depend on **v** and **a**. As mentioned earlier, the optimization is carried out using the toolboxes of MATLAB. Detailed modeling approach and schematic is highlighted in Fig. S1 (Supplementary Materials).

The presented scheme is general and it can accommodate –in principle– any question of interest related to solar cell design. This is done by identifying initially the main set of parameters to be optimized (**v**) and the fixed ones (**a**). To ensure that the optimized parameters are within acceptable physical ranges, **v** is bounded between lower **v**_*L*_ and upper **v**_*U*_ limits. Furthermore, additional constraints are imposed to ensure that the iterative variation of **v** allows proper device operation by forcing physical necessities such as band alignments. Also, more constraints can be added for other purposes. The objective function to be maximize is the efficiency; so,

1 $${\eta }_{\max }=\mathop{{\max }}\limits_{{{\bf{v}}}_{L} < {\bf{v}} < {{\bf{v}}}_{U}}\eta ({\bf{v}},{\bf{a}})$$

where *η* is

2 $$\eta =\frac{{V}_{oc}{J}_{sc}FF}{{P}_{in}},$$

*V*_*oc*_ is the open circuit voltage, *J*_*sc*_ is the short circuit current, *FF* is the fill factor, and *P*_*in*_ is AM1.5g input power. The optimization process continues iteratively till some predefined stopping criteria are satisfied. In this work, the stopping criteria is the convergence of the objective function within a tolerance of 10^−6^ whereas initial guesses are provided for each parameter within a physically acceptable range. Finally, the optimized vector **v** and the fixed one **a** compose the optimized material data set for each layer which can then be obtained by either material design or from the rich experimental data.

To select a suitable optimization algorithm for solar cells, seven different local and global optimization methods are compared by analyzing the speed of convergence to the maximum value of each method and by comparing these values. The used methods include a local gradient based method (Fmin)^39^, and three global methods; namely genetic algorithm (GA)^40^, particle swarm optimization (Pswarm)^41^ and pattern search (PattS)^42^. The other three employed optimizers are hybrid algorithms of the three global and the local optimizer to enhance the computational efficiency of the problem and the material data set.

For practical application, the full space device optimization procedure outlined above is explained in greater detail and applied for two problems; a) to find the physical properties for optimum ETM, HTM, and contacts and the layers thickness to maximize the efficiency of perovskites solar cell (PSC), and b) to identify different buffer layer materials for Cd-free CIGS solar cells.

### Application to Perovskite Solar Cells

In this subsection, the proposed method is applied to determine the optimum properties of ETM and HTM materials along with front and back contacts and the optimum layers’ thicknesses to maximize the efficiency of PSC. The device structure of PSC comprises of arbitrary contacts, ETM, HTM and CH_3_NH_3_PbI_3_ perovskite as an absorber (please see Fig. 1). There are 23 design parameters that should be optimized (listed below), which are combined in **v**. The crucial parameter of the perovskite absorber is its thickness, which is considered to optimize the performance as the thickness should be optimal to balance the carriers’ generation by its known absorption and their recombination. Two cases are considered; in the first one, only the intrinsic recombination properties are considered (without defects). In the second case, defects are introduced based on the minimal reported values in the literature. The reported values for the trap density vary widely^43^ due to the difficulty of measuring the trap density separately. Actually, the measurements (mostly based on photoluminescence experiments) determine the product of the trap density and capture cross section. Based on the methods and materials used for perovskite film growth, the reported trap densities are ranged between 10^8^–10^15^ cm^−3^ ^44–47^. Therefore, we assume a neutral defect at intrinsic Fermi level with trap density of 10^14^ cm^−3^ and capture cross section of 10^−14^ cm^2^.

The parameters to be optimized are:

- **Front and back contacts:** work function,
- **ETM & HTM layers:** dielectric permittivity, electron mobility, hole mobility, acceptor and donor concentration, band gap, the coefficients of the used absorption model (Eq. 3), conduction and valence bands densities, affinity energy, and thickness,
- **Perovskite absorbing layer:** thickness.

where the used absorption model for unknown absorption is

3 $$\alpha (\hslash \omega )=(A+\frac{B}{\hslash \omega })\sqrt{\hslash \omega -{E}_{g}}.$$

For the absorbing CH_3_NH_3_PbI_3_ properties, reported experimental values are used as shown in Table 1.

**Table 1:** The properties of CH_3_NH_3_PbI_3_.

Material properties	CH_3_NH_3_PbI_3_
Bandgap (eV)	1.5 ^70^
Electron affinity (eV)	3.9 ^71^
Dielectric permittivity	10 ^29^
Conduction band density of states (cm^−3^)	3.9 × 10^18^ ^29^
Valence band density of states (cm^−3^)	2.7 × 10^18^ ^29^
Electron mobility *μ*_*n*_ (cm^2^/V s)	2 ^72^
Hole mobility *μ*_*p*_ (cm^2^/V s)	2 ^72^
Donor density (cm^−3^)	10^9^ ^29^
Acceptor density (cm^−3^)	10^9^ ^29^
Electron thermal velocity (cm/s)	10^7^ ^29^
Hole thermal velocity (cm/s)	10^7^ ^29^
Radiative recombination coefficient (cm^3^/s)	2.3 × 10^−9^ ^29^

The absorption spectrum is extracted from reference^29^. Concerning the contacts, ideal ohmic is designated for front and back contacts with surface recombination velocity of 10^7^ cm/s. All the simulations are conducted assuming AM1.5g solar spectrum and at a temperature of 300 K.

To ensure the proper operation of the cell based on band alignment, the following constraints are applied in the simulation:

4 $${\chi }_{ETM}-{{\rm{\Phi }}}_{FC}\le 0,$$

5 $${\chi }_{HTM}-{\chi }_{P}\le 0,$$

6 $${{\rm{\Phi }}}_{BC}-{\chi }_{HTM}-{E}_{g,HTM}\le 0,$$

7 $${\chi }_{HTM}+{E}_{g,HTM}-{\chi }_{P}-{E}_{g,P}\le 0.$$

where *χ* is electron affinity, Φ is contact work function, and *E*_*g*_ is the bandgap while the subscripts P, FC, and BC stand for perovskite, front contact, and back contact respectively. The results are presented in the next section.

### Application to CIGS Solar Cells

**Figure 3:**
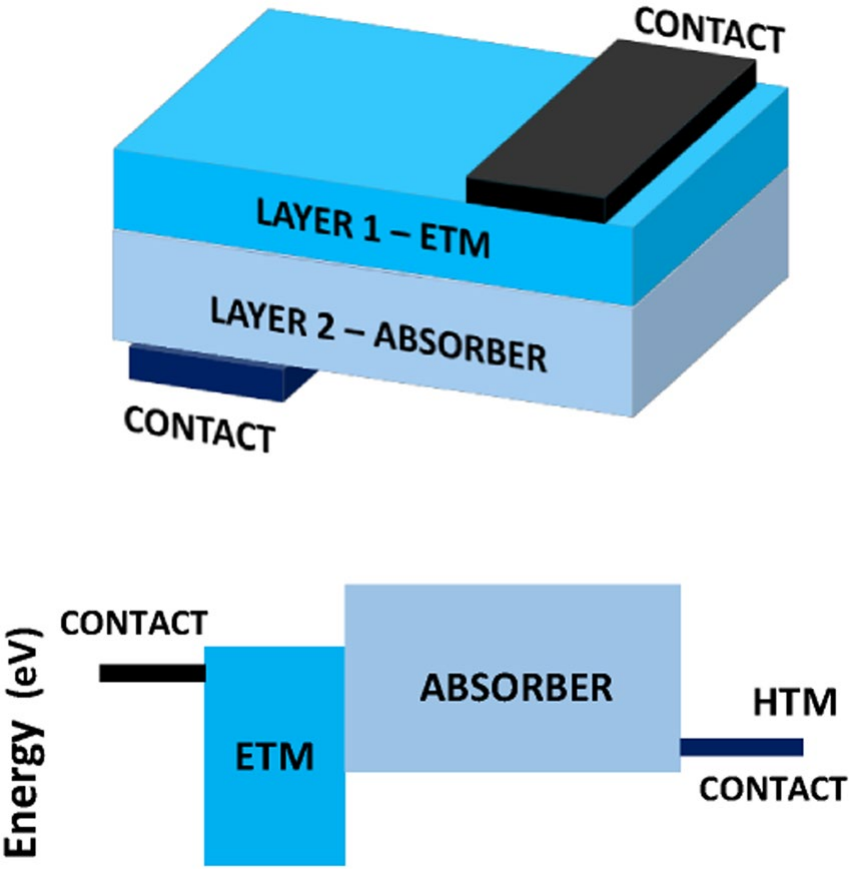
The considered structure of CIGS with single ETM layer.

Most of the designs of CIGS solar cells use cadmium sulfide CdS as buffer layer sandwiched between the *n*-type window (mostly ZnO or TiO_2_) and the CIGS absorbing layer. As CdS is not the absorber, it is auxiliary and could be replaced to avoid its toxicity and to have Cd-free CIGS solar cells. This has been tackled intermittently^48–52^. Here, we try to determine the needed properties of an alternative single ETM layer to make efficient Cd-free CIGS solar cells. The considered device structure is shown in Fig. 3. It is composed of front contact, ETM, *p*-CIGS, and back contact and without the typical CdS buffer layer stacking.

In the analysis, we consider two cases. In the first, the thickness of CIGS layer is fixed to 2.5 *μ*m as the commonly used nominal value. In the second, we optimize the thickness as well. Hereunder listed are the other parameters to be optimized:

- **Front and back contacts:** work function,
- **ETM:** dielectric permittivity, electron mobility, hole mobility, acceptor and donor concentration, band gap, the coefficients of the used absorption model (Eq. 3), conduction and valence bands densities, affinity energy, and thickness,
- **CIGS layer:** thickness.

For the absorbing CIGS properties, reported experimental values are used as shown in Table 2. The defects are assumed to be at intrinsic Fermi level (*E*_*F*_) with 10^14^ cm^−3^ trap density and capture cross section of 10^−14^ cm^2^ following deep-level transient spectroscopy results from references^53–56^. The absorption spectrum is extracted from reference^57^. As in the case of PSC, ideal ohmic contact is assumed for both front and back contacts with surface recombination velocity of 10^7^ cm/s. Also, all the simulations are conducted in AM1.5g solar spectrum and at a temperature of 300 K. To ensure a proper operation, the following constraint is applied:

8 $${\chi }_{ETM}-{{\rm{\Phi }}}_{FC}\le 0.$$

The results are presented in the next section.

**Table 2:** The properties of CIGS.

Material properties	CIGS
Bandgap (eV)	1.16 ^57^
Electron affinity (eV)	4.2 ^73^
Dielectric permittivity	13.6 ^73^
Conduction band density of states (cm^−3^)	2.2 × 10^18^ ^74^
Valence band density of states (cm^−3^)	1.8 × 10^18^ ^74^
Electron mobility *μ*_*n*_ (cm^2^/V s)	100 ^73^
Hole mobility *μ*_*p*_ (cm^2^/V s)	25 ^73^
Donor density (cm^−3^)	10 ^73^
Acceptor density (cm^−3^)	10^16^ ^73^
Electron thermal velocity (cm/s)	10^7^ ^32^
Hole thermal velocity (cm/s)	10^7^ ^32^
Radiative recombination coefficient (cm^3^/s)	2.0 × 10^−9^ ^29^

## Results and Discussion

### Comparison between optimization algorithms

Generally, the complexity of optimization problems vary significantly. So, what is suitable for a problem may not be suitable for another one. This depends on many factors and mainly the governing physical models and the numerical nature of the considered problem. In this work, the computational complexity is determined by the number of parameters to be optimized and the connectedness between them. In PSC optimization problem, 23 parameters are used while 13 are used for CIGS one. The numerical robustness of the proposed method has been validated by employing various local and global space search optimization algorithms for the two PSC case studies; namely PSC with and without defects. The validation shall ensure that the optimized material data set is a global solution and it allows us to assess the optimization efficiency of each optimization approach. This is done by analyzing and comparing the maximum obtained solar cell efficiencies and the computational time (estimated by the number of function counts).

**Figure 4:**
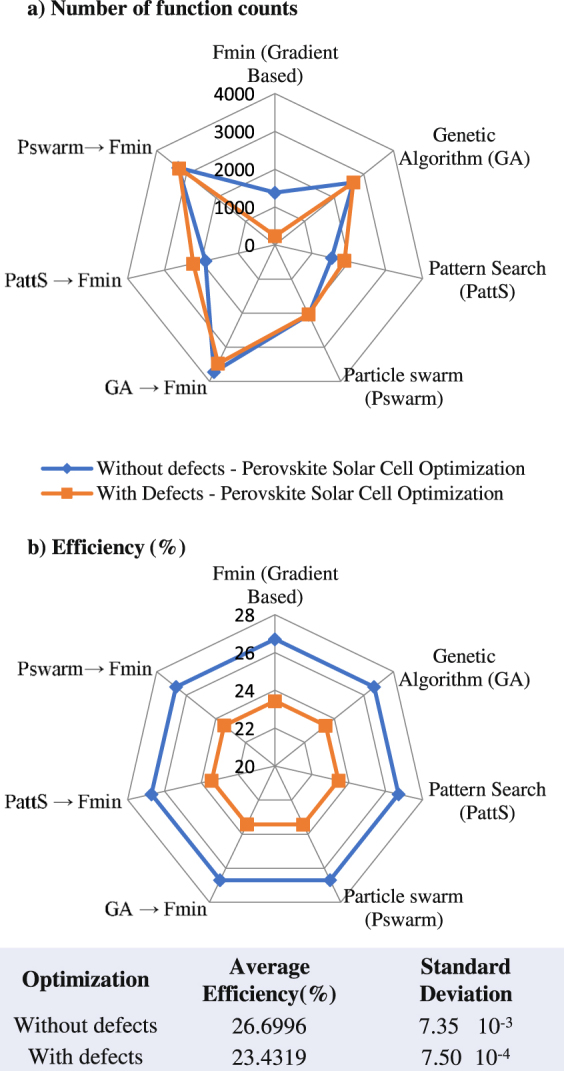
The computational cost and maximum conversion efficiencies of the used space search optimization method for the two PSC case studies.

Figure 4 shows the computational costs of all the used optimization methods to maximize the cell efficiency of PSC with and without defects and the obtained efficiency by each method. Clearly, the gradient based optimizer (Fmin) performed significantly better than all other methods in terms of computational cost while almost the same cell efficiency is obtained by all optimizers (26.6% for PSC without defects and 23.4% for PSC with defects). This is mainly due to the smoothness and continuity of the governing physical models and convex nature of the objective function (i.e. *η* (**v**, **a**)).

Fmin algorithm requires only 238 function count for PSC with defects and 1384 function count for PSC without defects. For the case of PSC optimization with non-intrinsic defects, the second fastest algorithm is pattern search which requires 1881 functions count while the slowest was the hybrid general algorithm and Fmin with 3478 function count. This is also observed for the case without non-intrinsic defects, pattern search needs 1538 function count while the hybrid general algorithm and Fmin algorithm uses 3729 function count. However, the absolute standard deviation of cell efficiencies obtained by all algorithms was 0.00735 and 0.00075 for PSC optimization with and without non-intrinsic defects,respectively. This illustrates that all the used local and global optimizers converge almost to the same value. However and as known, global optimizers yielded in slower convergence.

### The optimization of PSC design

Commonly, the main cause of the reduced efficiency of matured solar cells below the theoretical limit is the drop in the estimated *V*_*oc*_ while usually the obtained *J*_*sc*_ is around the theoretically maximum values^11,58,59^. For example in Si solar cell, the achieved *J*_*sc*_ is 41.8 mA/cm^2^ ^60^ while the maximum theoretically estimated value for 1.12 eV energy gap is 42.71 mA/cm^2^ ^58,59^. For CdTe solar cell, the achieved *J*_*sc*_ is 30.29 mA/cm^2^ ^1^ while the expected theoretical value for 1.45 eV energy gap is 30.54 mA/cm^2^ ^58,59^. As for PSC, the best reported and certified *J*_*sc*_ is 24.67 mA/cm^2^ ^61^ which is considerably less than the theoretical value of 29.51 mA/cm^2^ ^58,59^. This is mainly due to the reduced thickness of the absorber layer in PSC to mitigate the effects of non-radiative recombinations^62^. In principle, such effects are due to the reduced crystal quality and can be mitigated by improving the growth process quality. In other words, such effects are non-intrinsic and can be marginalized and hence they don’t dictate the practical limit of PSC conversion efficiency. In this subsection, we estimate the practical limit of it when the non-radiative recombinations are suppressed.

The details of the full space optimization implementation for PSC was explained in the Subsection entitled “Application to Perovskite Solar Cells”, where it is used to identify the optimum properties and thicknesses of contacts, HTM, ETM, and absorber layers that shall maximize the cell efficiency. The obtained data sets depend on the used optimizers and yielded different combination of values within physically acceptable range as shown by Tables S1 and S2 (Supplementary Materials). This is expected as some of the considered parameters shall result in extremely comparable cell performance within wide ranges. However, some of the parameters shall converge either individually or collectively. This variety of combinations is beneficial from the practical perspective of material screening for potential solar cell materials as they provide a window for device optimization parameters by selecting appropriate descriptors.

In both cases, with and without defects, the important parameter of the absorber layer thickness converged to average values 732 nm and 1104 nm with standard deviations from different optimizers of 2.46 nm and 4.6 nm, respectively. The thickness obtained for the case with defects is slightly more that those reported in literature^63^. It suggests that a good quality absorber with a thickness around 732 nm is needed to optimize PSC design if we consider the least reported defects. Also, the results predict the optimal energy gaps and electron affinity of ETM and HTM to optimize the band offsets and to maintain optical transparency into the absorber layer. Furthermore, it is implied that the hole mobility of HTM and the electron mobility of ETM should be high as their optimized values tend to be on the higher limits of their ranges.

**Figure 5:**
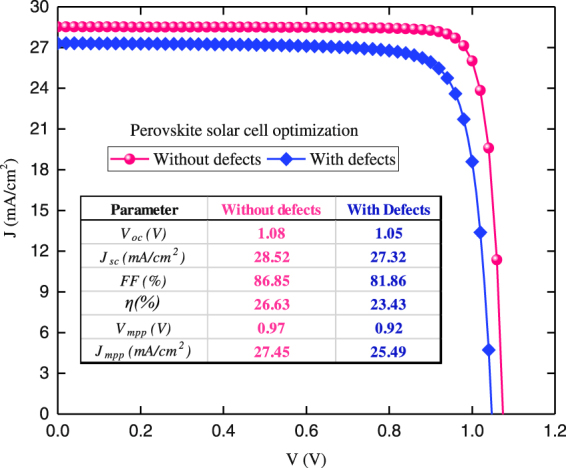
Optimized *J-V* curves for full space optimized PSC with and without defects. The design space covers 23 parameters for five layers i.e: contact/ETM/Absorber/HTM/contact that were optimized.

Figure 5 shows the *J*-*V* curves for the optimal PSC with and without defects as predicted by the best optimized material data set. They can realize conversion efficiencies of 26.6% without defects and 23.4% with defects under AM1.5g spectrum. For the case of defect-free, *V*_*oc*_ was found to be 1.07 V whereas *J*_*sc*_ is estimated to be 28.5 mA/cm^2^. By considering the defects in perovskite layer, both *V*_*oc*_ and *J*_*sc*_ are slightly reduced to 1.04 V and 27.3 mA/cm^2^ respectively. However, *FF* is considerably affected as it is reduced from 86.85% to 81.86%. In comparison with the experimentally reported values, *V*_*oc*_ and *FF* are in agreeable range with the resulted optimized values. However, there is a room to improve *J*_*sc*_. Practically, this can be achieved by careful engineering of growth quality, maximum charge extraction, and minimum recombination for transport mediums.

**Figure 6:**
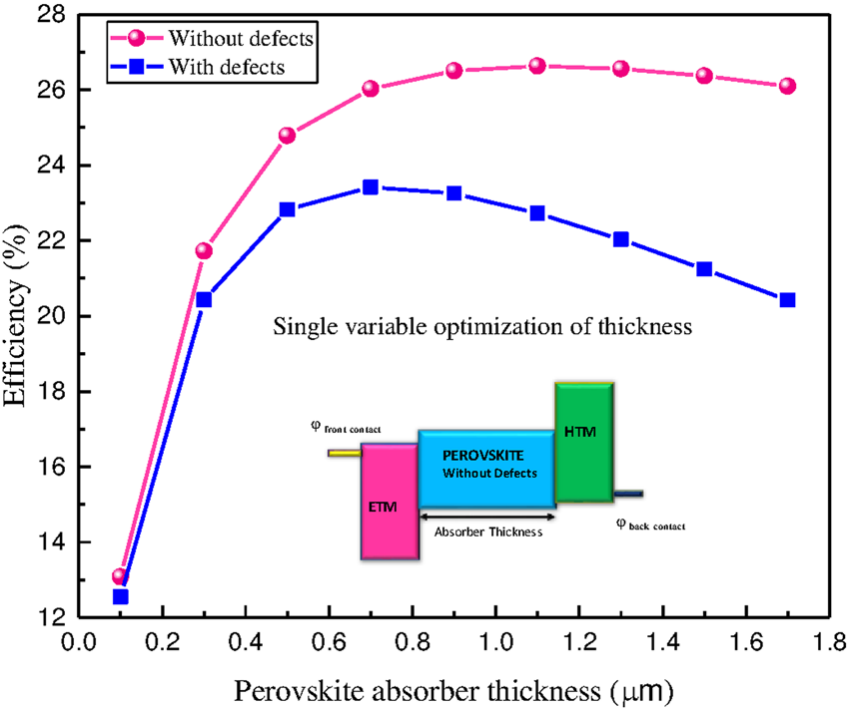
Single variable optimization of thickness for PSC with and without defects by keeping other full space optimized parameters fixed.

The next analysis for PSC is the issue of absorber thickness. As discussed above, the reported thicknesses are less than what is needed to optimize *J*_*sc*_. By fixing the obtained optimal parameters and varying only the absorbing layer thickness, *J*_*sc*_ peaks. Below the peak, *J*_*sc*_ is limited by the reduced absorption due to the small thickness. Above the peak, the non-radiative recombination becomes the major process that limits *J*_*sc*_. This is clearly illustrated in the results shown in Fig. 6. For PSC without defects, 26.6% conversion efficiency can be attained around 1.1 *μ*m whereas for PSC with defects 23.4% conversion efficiency can be achieved by an absorber thickness of 0.7 *μ*m under AM1.5g spectrum. Therefore, the resulted optimum thickness is a strong function of the quality of the material and absorption spectrum of the active layer.

**Figure 7:**
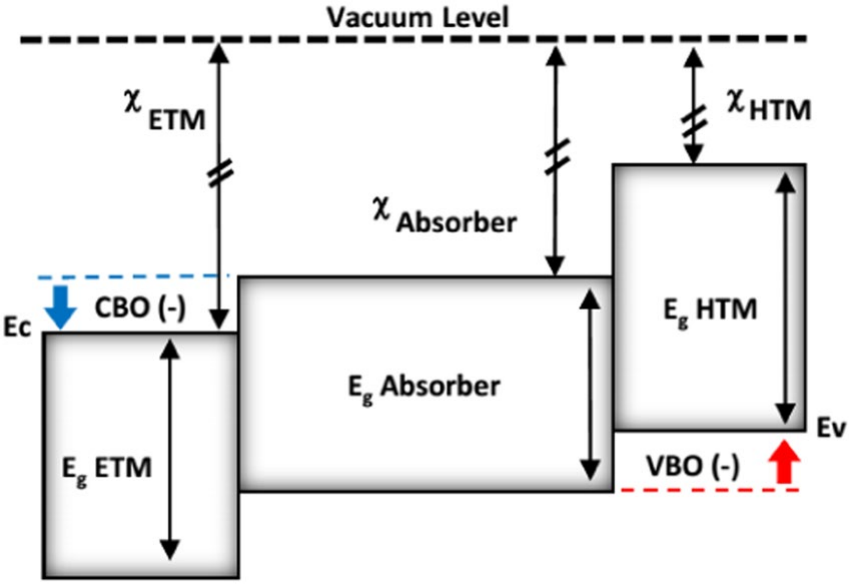
Band alignments of ETM/Absorber/HTM stack with Conduction Band Offset (CBO) and Valence Band Offset (VBO) for energy cliff operation.

The last analysis in this subsection is to investigate the effects of the band offsets between the perovskite layer and both ETM (conduction band offset CBO) and HTM (valence band offset VBO) layers as schematically shown in Fig. 7. This is done by fixing all the parameters and vary only electron affinities of both ETM and HTM layers (i.e. reduce the dimension of **v** to 2). This illustrates the the flexibility and robustness of the presented full space optimization method. It would be expected that by increasing the offsets, *J*_*sc*_ would basically increase and *V*_*oc*_ would decrease. However, there are many other associated issues as discussed shortly. So, it is essential to find the optimize offsets that maximize the power (i.e. the product of the voltage and the current)^64–67^.

By fixing the band gaps and varying electron affinity, band alignment changes and hence dictates the transport properties of photo-generated carriers^68^. The considered band offsets are only those that allow a proper operation of the cell and don’t form barriers^33^.

This is imposed by the following constraints:

9 $${\rm{\Delta }}{E}_{c}=-({\chi }_{ETM}-{\chi }_{P})\le 0,$$

and

10 $${\rm{\Delta }}{E}_{v}=-({\chi }_{P}+{E}_{g,P}-{\chi }_{HTM}-{E}_{g,HTM})\le 0.$$

All the other parameters are fixed according to the optimized data set using the gradient based Fmin method (Tables S1 and S2 in Supplementary Materials).

**Figure 8:**
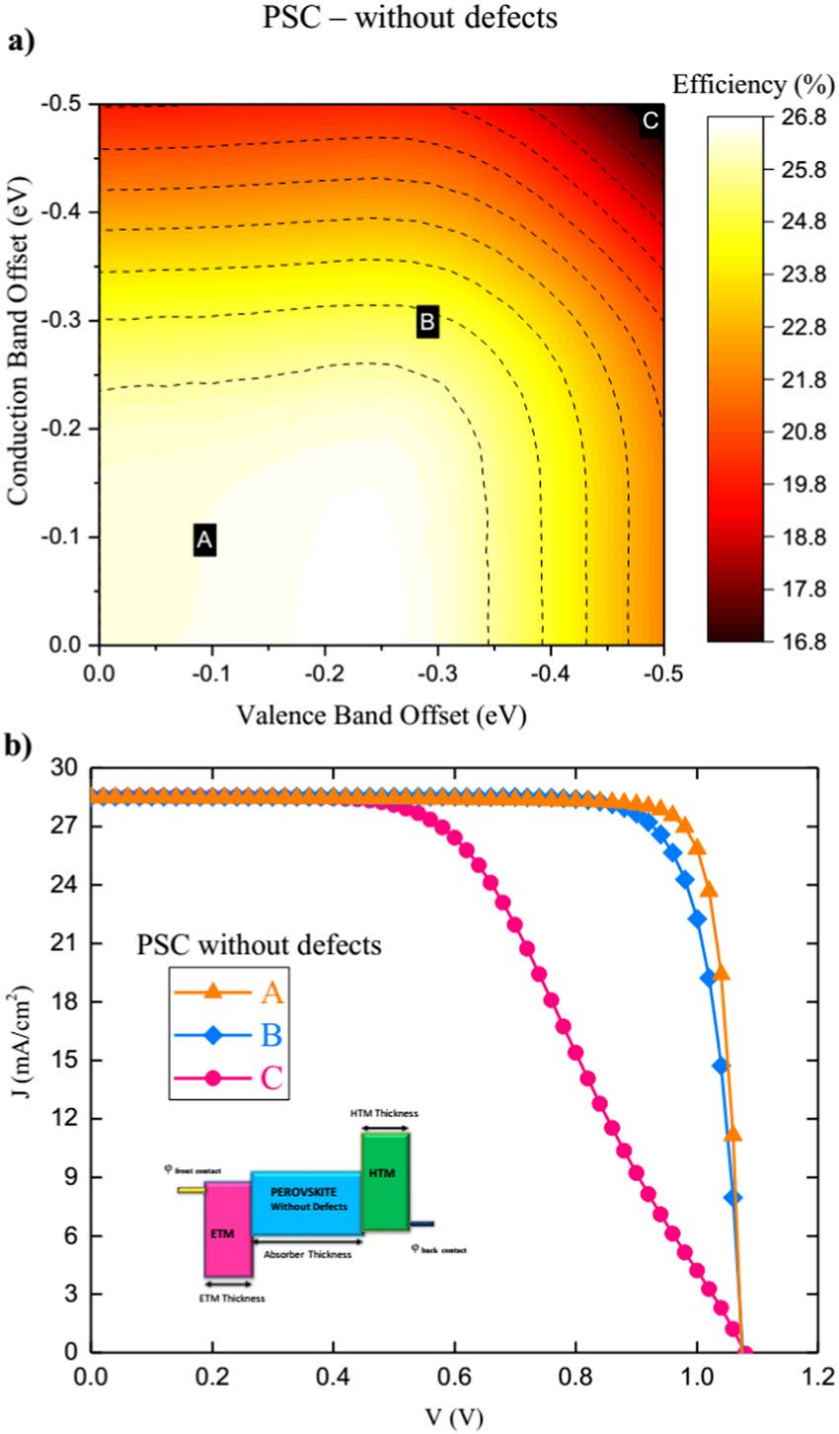
PSC without defects (**a**) Performance contours of efficiency for PSC without defects optimized by varying CBO and VBO and (**b**) *J-V* curves for characteristics points A,B and C on performance contours.

**Figure 9:**
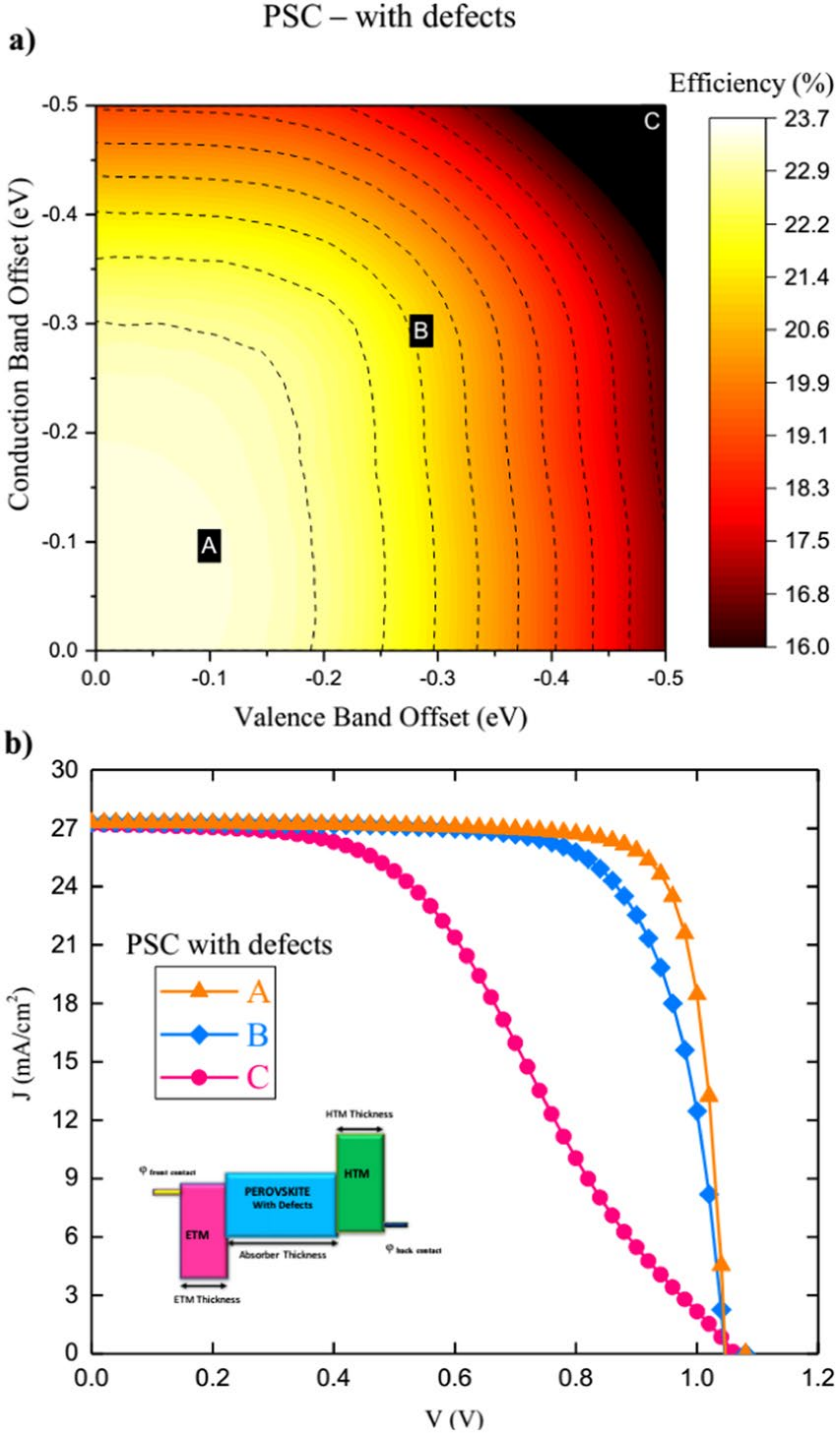
PSC with defects (**a**) Performance contours of efficiency for PSC with defects optimized by varying CBO and VBO and (**b**) *J-V* curves for characteristic points A,B and C on performance contours.

The resulted efficiencies are represented vs. CBO and VBO in the contour graphs (Figs 8a and 9a) for the cases of without and with defects respectively. The representative *J*-*V* curves corresponding to points A, B, and C on the contour plots are as shown as well in Figs 8b and 9b. The contours identify the optimum band offsets for both interfaces. As all the other parameters are fixed, the maximum obtained efficiencies of 23.4% and 26.6% for both cases, as in full-space optimization. They are corresponding to small CBO and VBO values as expected^33^. Band offset is vital for the charge transport and extraction as the barrier height determines the contact resistance. The performance gets reduced gradually with both offsets. However, rate of reduction depends the properties of ETM and HTM. For example, the effect of CBO is more dominant for PSC without defects while for PSC with defects, the effect of VBO is more dominant. This is related to density of states of ETM and HTM which further governs Fermi level (*E*_*F*_) and band bending at the interfaces.

### Cd-free CIGS solar cells

**Figure 10:**
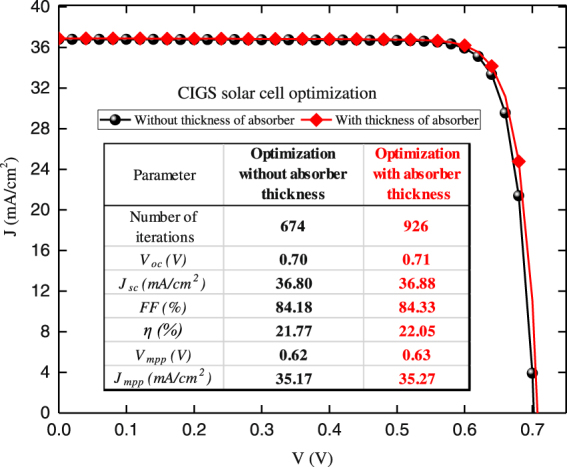
The optimized *J-V* curves for full space optimized CIGS with and without considering absorber thickness. The design space covers 13 parameters for contact/ETM/absorber.

As discussed in the Subsection entitled “Application to CIGS Solar Cells”, there is a growing interest to develop Cd-free CIGS solar cells. There, we discussed the two considered optimization problems. In the first one, the thickness of CIGS layer is fixed to 2500 nm while in the second, it is optimized as well. The resulted *J*-*V* curves of the optimized properties and thickness of ETM layer are shown in Fig. 10. After the initial validation of algorithms, gradient based optimizer was employed for two cases. The optimizer needed only 674 and 926 function counts to reach to the optimized structures.

The method identifies the optimized properties of ETM layer in both cases with and without considering CIGS thickness as shown in Table S3 (Supplementary Materials). The analysis showed that the maximum efficiency of 21.77% can be achieved by just a single buffer layer having the estimated optimized parameters. Moreover, if the thickness of CIGS absorber is optimized, its efficiency is increased to 22.05% where the absorber thickness was found to be 3.9 *μ*m.The value obtained is a strong function of the absorption spectrum employed and the type of grading in the absorber material. The calculated *V*_*oc*_ are 0.70 V and 0.71 V for Cd-free CIGS without and with absorber thickness optimization. *J*_*sc*_ is increased slightly as well from 36.80 to 36.88 mA/cm^2^ by including thickness optimization.

**Figure 11:**
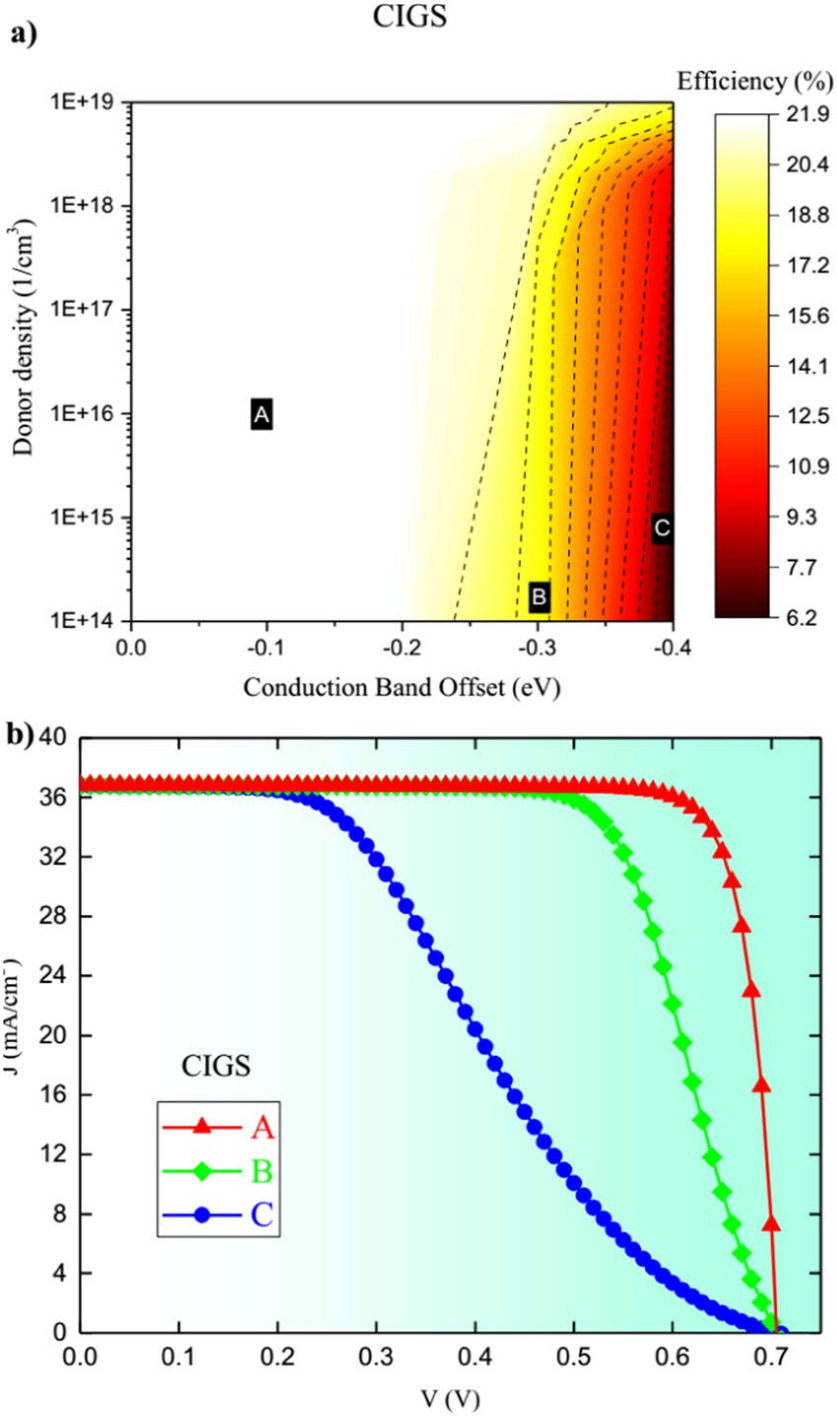
CIGS (**a**) Performance contours of efficiency for CIGS for varying CBO and donor density and (**b**) *J-V* curves for characteristic points A,B and C on performance contours.

After identifying the optimal properties of ETM layer, we investigate the effects of CBO and donor density on the electron injection from CIGS to ETM and hence the efficiency. CBO must be negative to avoid having barrier at the interface. Moreover, the main factor that could cause a complication is the position of Fermi level (*E*_*F*_) in ETM which is associated with the donor density. Figure 11 shows (a) the obtained cell efficiency vs. CBO and donor density and (b) *J*-*V* curves corresponding to points A, B, and C on the contour plots. Donor density was varied from 10^14^ to 10^19^ whereas CBO was varied from 0.0 to −0.4 eV by changing electron affinity. Clearly, the performance is improved with the increased donor density which reduces the effect of CBO for highly doped ETM as shown by the full space optimized donor density of 7.94 × 10^18^. However, if the doping density is reduced, CBO starts playing an important negative role and hence it must be kept small.

**Figure 12:**
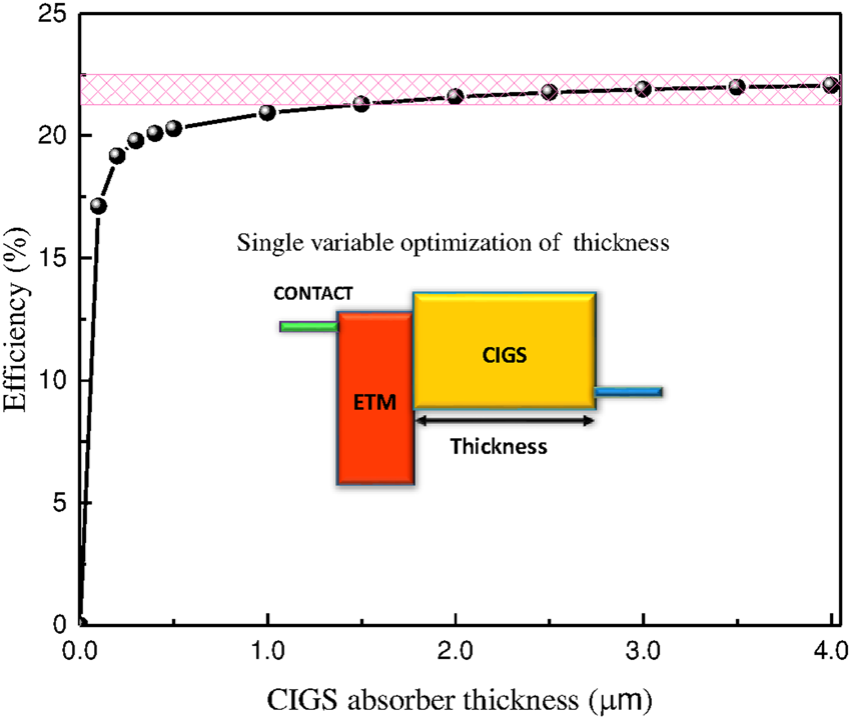
The conversion efficiency of CIGS solar cell vs. the absorber thickness.

The effect of thickness for CIGS layer is studied by varying it while maintaining the other identified optimum parameters for ETM and the resulted efficiency is shown in Fig. 12. Clearly, the efficiency increases with the thickness. Eventually, it will start decreasing once the recombination becomes influential. Within the used range, the maximum efficiency of 22.04% is obtained at the higher thickness range of 4.0 *μ*m; but, it would be practical to limit the thickness to something around 2.0 *μ*m while a tiny reduction of the efficiency, which is 21.59% at this thickness. This small efficiency reduction is displayed by shaded rectangle in Fig. 12 is in accordance with experimental values^69^. Lastly, the obtained optimum properties of ETM layer shall allow us to identify alternative non-toxic ETMs that can improve CIGS solar cells.

## Conclusion

Multi-property solar cell device optimization is developed and applied. It provides a comprehensive design optimization framework for solar cells and can be extended to any solid-state device by avoiding the recurrent paradigms in the solar cell designs. The approach couples a drift-diffusion solver of solar cells with several evolutionary and non-evolutionary optimization algorithms. This results in identifying the properties of the optimal matching materials for a given absorber by selecting a set of different parameters for different layers of solar cells. The method is implemented for two state of the art solar cell designs problems; namely to optimize the design of PSC, and to have Cd-free CIGS solar cell.

Numerically, many local and global optimizers were employed and it was found that gradient based methods perform best due to the smoothness of physical models of solar cell and objective function. From the simulations, we found that PSC can reach an efficiency of 26.6% with defects and 23.4% with defects can be achieved by deploying the predicted material data set for ETM, HTM, and contacts. Simulations from non-toxic CIGS solar cell identify the needed properties for ETM layer to maximize the efficiency to around 22.04%.

## Electronic supplementary material

Supplementary Material

## References

[1] Green MA (2017). Solar cell efficiency tables (version 49). Prog. Photovoltaics: Res. Appl..

[2] Gloeckler M, Sankin I, Zhao Z (2013). CdTe solar cells at the threshold to 20% efficiency. IEEE Journal of Photovoltaics.

[3] Morales-Acevedo A (2006). Can we improve the record efficiency of CdS/CdTe solar cells?. Solar energy materials and solar cells.

[4] Reinhard, P. *et al*. Review of progress toward 20% efficiency flexible cigs solar cells and manufacturing issues of solar modules. In *Photovoltaic Specialists Conference (PVSC), Volume 2, 2012 IEEE 38th*, 1–9 (IEEE, 2012).

[5] Meyer B, Klar P (2011). Sustainability and renewable energies–a critical look at photovoltaics. physica status solidi (RRL)-Rapid Research Letters.

[6] Jackson P (2016). Effects of heavy alkali elements in Cu (In,Ga)Se_2_ solar cells with efficiencies up to 22.6%. physica status solidi (RRL)-Rapid Research Letters.

[7] Green MA, Bremner SP (2017). Energy conversion approaches and materials for high-efficiency photovoltaics. Nature Materials.

[8] Pandey A (2016). Recent advances in solar photovoltaic systems for emerging trends and advanced applications. Renewable and Sustainable Energy Reviews.

[9] Armaroli N, Balzani V (2016). Solar electricity and solar fuels: status and perspectives in the context of the energy transition. Chemistry–A European Journal.

[10] Ramanujam J (2016). Inorganic photovoltaics-planar and nanostructured devices. Progress in Materials Science.

[11] Hossain M, Alharbi F (2013). Recent advances in alternative material photovoltaics. Materials Technology.

[12] McFarland E (2014). Solar energy: setting the economic bar from the top-down. Energy & Environmental Science.

[13] Andersson BA (2000). Materials availability for large-scale thin-film photovoltaics. Prog. Photovoltaics: Res. Appl..

[14] Ghosh A, Krishnan Y (2014). At a long-awaited turning point. Nature nanotechnology.

[15] Gorjiara T, Baldock C (2014). Nanoscience and nanotechnology research publications: a comparison between australia and the rest of the world. Scientometrics.

[16] Alharbi F (2011). Abundant non-toxic materials for thin film solar cells: Alternative to conventional materials. Renewable Energy.

[17] Green MA (2005). Silicon photovoltaic modules: a brief history of the first 50 years. Prog. Photovoltaics: Res. Appl..

[18] Goetzberger A, Hebling C, Schock H-W (2003). Photovoltaic materials, history, status and outlook. Materials Science and Engineering: R: Reports.

[19] Yamaguchi K, Nakayama N, Matsumoto H, Ikegami S (1977). CdS-CdTe solar cell prepared by vapor phase epitaxy. Japanese Journal of Applied Physics.

[20] Shay J, Wagner S, Kasper H (1975). Efficient CuInSe_2_/CdS solar cells. Applied Physics Letters.

[21] Oakes J, Greenfield I, Partain L (1977). Diffusion length determination in thin-film C_u*x*_S/CdS solar cells by scanning electron microscopy. Journal of Applied Physics.

[22] Wagner S, Shay J, Bachmann K, Buehler E (1975). p-InP/n-CdS solar cells and photovoltaic detectors. Applied Physics Letters.

[23] Roy P, Berger S, Schmuki P (2011). TiO_2_ nanotubes: synthesis and applications. Angewandte Chemie International Edition.

[24] Butler KT, Frost JM, Skelton JM, Svane KL, Walsh A (2016). Computational materials design of crystalline solids. Chemical Society Reviews.

[25] Brandt, R. E. *Accelerating the development of novel photovoltaic materials*. Ph.D. thesis, Massachusetts Institute of Technology (2016).

[26] Bernardi M, Grossman JC (2016). Computer calculations across time and length scales in photovoltaic solar cells. Energy & Environmental Science.

[27] Grånäs, O., Vinichenko, D. & Kaxiras, E. Establishing the limits of efficiency of perovskite solar cells from first principles modeling. *Scientific Reports* **6** (2016). 10.1038/srep36108 PMC5099916 27824030

[28] Rondinelli JM, Poeppelmeier KR, Zunger A (2015). Research update: Towards designed functionalities in oxide-based electronic materials. APL Materials.

[29] Hossain MI, Alharbi FH, Tabet N (2015). Copper oxide as inorganic hole transport material for lead halide perovskite based solar cells. Solar Energy.

[30] Yadav P (2015). Exploring the performance limiting parameters of perovskite solar cell through experimental analysis and device simulation. Solar Energy.

[31] Liu F (2014). Numerical simulation: toward the design of high-efficiency planar perovskite solar cells. Applied Physics Letters.

[32] Minemoto T, Murata M (2014). Device modeling of perovskite solar cells based on structural similarity with thin film inorganic semiconductor solar cells. Journal of applied physics.

[33] Minemoto T, Murata M (2015). Theoretical analysis on effect of band offsets in perovskite solar cells. Solar Energy Materials and Solar Cells.

[34] Altermatt PP, Kiesewetter T, Ellmer K, Tributsch H (2002). Specifying targets of future research in photovoltaic devices containing pyrite (FeS2) by numerical modelling. Solar energy materials and solar cells.

[35] Jovanovic R, Kais S, Alharbi FH (2014). Cuckoo search inspired hybridization of the nelder-mead simplex algorithm applied to optimization of photovoltaic cells. Applied Mathematics & Information Sciences.

[36] Matlab, optimization toolbox, and global optimization toolbox release 2016a. Tech. Rep., The MathWorks Inc., Natick, MA, US (2016).

[37] Burgelman M, Nollet P, Degrave S (2000). Modelling polycrystalline semiconductor solar cells. Thin Solid Films.

[38] Burgelman M, Verschraegen J, Degrave S, Nollet P (2004). Modeling thin-film pv devices. Prog. Photovoltaics: Res. Appl..

[39] Byrd RH, Gilbert JC, Nocedal J (2000). A trust region method based on interior point techniques for nonlinear programming. Mathematical Programming.

[40] Goldberg, D. E. *Genetic Algorithms in Search, Optimization and Machine Learning*, 1st edn, (Addison-Wesley Longman Publishing Co., Inc., Boston, MA, USA, 1989).

[41] Kennedy, J. Particle swarm optimization. In *Encyclopedia of machine learning*, 760–766 (Springer, 2011).

[42] Audet C, Dennis JE (2002). Analysis of generalized pattern searches. SIAM Journal on Optimization.

[43] Leijtens T (2016). Carrier trapping and recombination: the role of defect physics in enhancing the open circuit voltage of metal halide perovskite solar cells. Energy Environ. Sci..

[44] Heo, S. *et al*. Deep level trapped defect analysis in CH3NH3PbI3 perovskite solar cells by deep level transient spectroscopy. *Energy Environ. Sci*. 10.1039/C7EE00303J (2017).

[45] Shi D (2015). Low trap-state density and long carrier diffusion in organolead trihalide perovskite single crystals. Science.

[46] Saidaminov MI (2015). High-quality bulk hybrid perovskite single crystals within minutes by inverse temperature crystallization. Nature Communications.

[47] Lian Z (2016). Perovskite CH3NH3PbI3(Cl) single crystals: Rapid solution growth, unparalleled crystalline quality, and low trap density toward 108 cm^−3^. Journal of the American Chemical Society.

[48] Ohtake Y, Okamoto T, Yamada A, Konagai M, Saito K (1997). Improved performance of Cu(InGa)Se_2_ thin-film solar cells using evaporated Cd-free buffer layers. Solar Energy Materials and Solar Cells.

[49] Ennaoui A, Siebentritt S, Lux-Steiner MC, Riedl W, Karg F (2001). High-efficiency Cd-free cigss thin-film solar cells with solution grown zinc compound buffer layers. Solar Energy Materials and Solar Cells.

[50] Nakada T, Mizutani M (2002). 18% efficiency Cd-free Cu(In, Ga)Se_2_ thin-film solar cells fabricated using chemical bath deposition (cbd)-ZnS buffer layers. Japanese Journal of Applied Physics.

[51] Friedlmeier TM (2015). Improved photocurrent in Cu(In, Ga)Se_2_ solar cells: from 20.8% to 21.7% efficiency with CdS buffer and 21.0% Cd-free. IEEE Journal of Photovoltaics.

[52] Hiroi H, Iwata Y, Adachi S, Sugimoto H, Yamada A (2016). New world-record efficiency for pure-sulfide Cu(In, Ga)S_2_ thin-film solar cell with cd-free buffer layer via kcn-free process. IEEE Journal of Photovoltaics.

[53] Heo, S. *et al*. Defect visualization of Cu(InGa)(SeS)_2_ thin films using dlts measurement. *Scientific Reports* **6** (2016). 10.1038/srep30554 PMC4967860 27476672

[54] Johnson P, Heath J, Cohen J, Ramanathan K, Sites J (2005). A comparative study of defect states in evaporated and selenized cigs (s) solar cells. Prog. Photovoltaics: Res. Appl..

[55] Song, S. *et al*. Structure optimization for a high efficiency cigs solar cell. In *Photovoltaic Specialists Conference (PVSC), 2010 35th IEEE*, 002488–002492 (IEEE, 2010).

[56] Pettersson J, Platzer-Björkman C, Zimmermann U, Edoff M (2011). Baseline model of graded-absorber cu (in, ga) se 2 solar cells applied to cells with zn 1- x mg x o buffer layers. Thin Solid Films.

[57] Sharbati S, Sites JR (2014). Impact of the band offset for n-Zn(O, S)/p-Cu(In, Ga)Se_2_ solar cells. IEEE Journal of Photovoltaics.

[58] Alharbi FH (2015). An efficient descriptor model for designing materials for solar cells. npj Computational Materials.

[59] Alharbi FH, Kais S (2015). Theoretical limits of photovoltaics efficiency and possible improvements by intuitive approaches learned from photosynthesis and quantum coherence. Renewable and Sustainable Energy Reviews.

[60] Masuko K (2014). Achievement of more than 25% conversion efficiency with crystalline silicon heterojunction solar cell. IEEE Journal of Photovoltaics.

[61] Yang WS (2015). High-performance photovoltaic perovskite layers fabricated through intramolecular exchange. Science.

[62] Johnston MB, Herz LM (2015). Hybrid perovskites for photovoltaics: Charge-carrier recombination, diffusion, and radiative efficiencies. Accounts of chemical research.

[63] Chiang, C.-H., Nazeeruddin, M. K., Grätzel, M. & Wu, C.-G. The synergistic effect of H_2_O and dmf towards stable and 20% efficiency inverted perovskite solar cells. *Energy & Environmental Science* (2017).

[64] Minemoto T (2001). Theoretical analysis of the effect of conduction band offset of window/cis layers on performance of cis solar cells using device simulation. Solar Energy Materials and Solar Cells.

[65] Gloeckler M, Sites J (2005). Efficiency limitations for wide-band-gap chalcopyrite solar cells. Thin Solid Films.

[66] Törndahl T, Platzer-Björkman C, Kessler J, Edoff M (2007). Atomic layer deposition of Zn1^−*x*^ Mg^*x*^ O buffer layers for Cu(In, Ga)Se_2_ solar cells. Prog. Photovoltaics: Res. Appl..

[67] Sinsermsuksakul P (2013). Enhancing the efficiency of SnS solar cells via band-offset engineering with a zinc oxysulfide buffer layer. Applied Physics Letters.

[68] Anderson R (1960). Germanium-gallium arsenide heterojunctions [letter to the editor]. IBM Journal of Research and Development.

[69] Pettersson J, Törndahl T, Platzer-Björkman C, Hultqvist A, Edoff M (2013). The influence of absorber thickness on Cu(In, Ga)Se_2_ solar cells with different buffer layers. IEEE Journal of Photovoltaics.

[70] Mei A (2014). A hole-conductor–free, fully printable mesoscopic perovskite solar cell with high stability. Science.

[71] Green MA, Ho-Baillie A, Snaith HJ (2014). The emergence of perovskite solar cells. Nature Photonics.

[72] Ponseca CS (2014). Organometal halide perovskite solar cell materials rationalized: ultrafast charge generation, high and microsecond-long balanced mobilities, and slow recombination. Journal of the American Chemical Society.

[73] Asaduzzaman M, Hasan M, Bahar AN (2016). An investigation into the effects of band gap and doping concentration on Cu(In, Ga)Se_2_ solar cell efficiency. SpringerPlus.

[74] Atourki L, Kirou H, Ihlal A, Bouabid K (2016). Numerical study of thin films cigs bilayer solar cells using scaps. Materials Today: Proceedings.

