# Supplementary material for: Full space device optimization for solar cells
Source: Sci Rep. 2017 Sep 20;7:11984. doi: 10.1038/s41598-017-12158-0 (PMC5607268; doi:10.1038/s41598-017-12158-0)
Supplement: Supplementary file 1 — Supplementary Material [file 41598_2017_12158_MOESM1_ESM.pdf]

Full Space Device Optimization for Solar Cells

Ahmer A.B. Baloch, Shahzada P. Aly, Mohammad I. Hossain, Fedwa El Mellouhi,
Nouar Tabet, and Fahhad H Alharbi

Supplementary Material

Supplementary Material

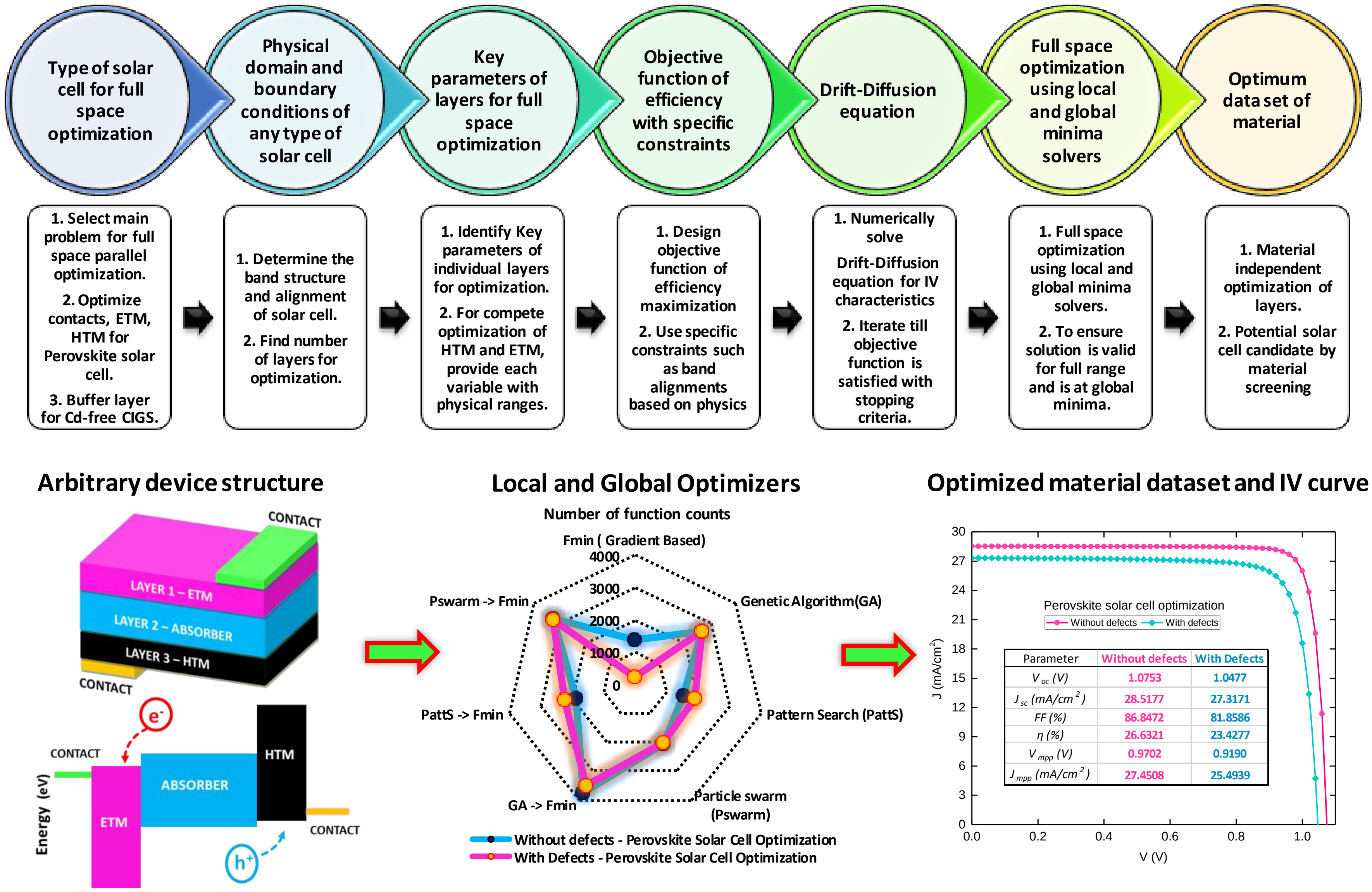

Figure S1: Detailed framework for full space optimization with schematics and results from simulation.

Table S1

Full space optimized parameters for PSC with Defects.

PSC with Defects		Optimization Algorithms						
**Variable**	**Unit**	**fmin**	**GA**	**PattS**	**Particle Swarm**	**GA to Fmin**	**PattS to Fmin**	**Pswarm to Fmin**
**Front Contact Work function**	eV	4.0964	3.9137	4.1000	4.0021	3.9170	4.1000	4.0021
**Back Contact Work function**	eV	5.1738	5.3478	5.2250	5.3900	5.3425	5.2250	5.3900
**Thickness ETM**	µm	0.1707	0.3906	0.0750	0.0500	0.3694	0.0750	0.0500
**Donor Conc. ETM**	$1/cm^3$ (10^{value})	18.5685	17.4060	18.5310	18.1480	17.3525	18.5310	18.1480
**Energy Gap ETM**	eV	3.9375	3.6517	4.0000	4.0000	3.6606	4.0000	4.0000
**Electron Mobility ETM**	$cm^2/V.s$	175.4944	1.4204	200.0000	233.9900	2.8493	200.0000	233.9900
**Hole Mobility ETM**	$cm^2/V.s$	5.4444	0.3584	1.0000	3.3803	0.2875	1.0000	3.3803
**Absorbtion Coeff. ETM**	$1/cm.eV^{1/2}$	125.7477	126.3281	125.8925	1557.0408	138.6756	125.8925	1557.0408
**CB DOS ETM**	$1/cm^3$ (10^{value})	18.5686	18.1680	18.5310	18.1640	18.1694	18.5310	18.1640
**VB DOS ETM**	$1/cm^3$ (10^{value})	18.5241	18.1210	18.0000	18.0880	18.0934	18.0000	18.0880
**Affinity Energy ETM**	eV	4.0303	3.9117	4.0000	3.9913	3.9091	4.0000	3.9913
**Dielectric Perm. ETM**	-	7.3402	5.6484	6.5000	6.0039	5.7548	6.5000	6.0039
**Thickness Perovskite**	µm	0.7321	0.7291	0.7324	0.7324	0.7375	0.7324	0.7324
**Thickness HTM**	µm	0.4989	0.0743	0.4500	0.5000	0.0742	0.4500	0.5000
**Acceptor Conc. HTM**	$1/cm^3$ (10^{value})	18.2066	18.0480	19.0000	18.0000	18.0672	19.0000	18.0000
**Energy Gap HTM**	eV	3.3645	2.8238	2.8996	2.9872	2.8235	2.8999	2.9872
**Electron Mobility HTM**	$cm^2/V.s$	4.9727	1.8524	2.0000	3.8813	1.5428	2.0000	3.8813
**Hole Mobility HTM**	$cm^2/V.s$	436.1325	155.8300	200.0000	230.8800	176.6236	200.0000	307.7459
**Absorbtion Coeff. HTM**	$1/cm.eV^{1/2}$	8529.0370	211.5437	1258.9254	1737.0007	213.8454	1258.9254	1737.0007
**CB DOS HTM**	$1/cm^3$ (10^{value})	19.9589	18.0610	18.0000	18.2750	18.0673	18.0000	18.2750
**VB DOS HTM**	$1/cm^3$ (10^{value})	18.2070	18.0580	19.0000	18.0000	18.0674	19.0000	18.0000
**Affinity Energy HTM**	eV	2.0355	2.5767	2.5000	2.4128	2.5765	2.5000	2.4128
**Dielectric Perm. HTM**	-	16.1612	4.9913	20.0000	20.0000	7.2553	20.0000	20.0000

Table S2

Full space optimized parameters for PSC without Defects.

**PSC without Defects**		**Optimization Algorithms**						
**Variable**	**Unit**	**fmin**	**GA**	**PattS**	**Particle Swarm**	**GA to Fmin**	**PattS to Fmin**	**Pswarm to Fmin**
**Front Contact Work function**	eV	4.0763	4.1142	4.1000	4.0763	4.0398	4.1000	4.0763
**Back Contact Work function**	eV	5.3759	5.1449	5.3500	5.3758	5.2561	5.3500	5.3758
**Thickness ETM**	µm	0.5000	0.1239	0.0594	0.5000	0.0703	0.0594	0.5000
**Donor Conc. ETM**	$1/cm^3$ (10^{value})	17.7791	16.3900	17.8280	17.7790	16.3652	17.8280	17.7790
**Energy Gap ETM**	eV	4.0000	3.3195	4.0000	4.0000	3.4842	4.0000	4.0000
**Electron Mobility ETM**	$cm^2/V.s$	191.3561	2.6214	200.0000	191.3800	19.4276	200.0000	191.3800
**Hole Mobility ETM**	$cm^2/V.s$	5.4391	4.0548	1.0000	5.4389	3.6442	1.0000	5.4389
**Absorbtion Coeff. ETM**	$1/cm.eV^{1/2}$	2.0000	2.2006	2.0375	2.0000	2.0277	2.0375	2.0000
**CB DOS ETM**	$1/cm^3$ (10^{value})	18.7489	19.4580	20.0000	18.7490	19.5973	20.0000	18.7500
**VB DOS ETM**	$1/cm^3$ (10^{value})	18.0379	19.0920	18.0000	18.0380	18.8936	18.0000	18.0380
**Affinity Energy ETM**	eV	4.0692	3.9378	4.0000	4.0692	3.9354	4.0000	4.0692
**Dielectric Perm. ETM**	-	8.3914	3.4762	19.0000	8.3917	3.8342	19.0000	8.3917
**Thickness Perovskite**	µm	1.1024	1.0993	1.1094	1.1017	1.1104	1.1094	1.1019
**Thickness HTM**	µm	0.4995	0.4425	0.4969	0.5000	0.4393	0.4969	0.4993
**Acceptor Conc. HTM**	$1/cm^3$ (10^{value})	17.9999	18.6960	19.0000	18.0000	18.7599	19.0000	18.0000
**Energy Gap HTM**	eV	3.3337	3.6016	2.8996	3.3337	3.6004	2.8996	3.3337
**Electron Mobility HTM**	$cm^2/V.s$	4.5182	0.2755	2.0000	4.5184	1.2107	2.0000	4.5184
**Hole Mobility HTM**	$cm^2/V.s$	361.0323	3.3577	200.0000	360.9900	3.7668	200.0000	360.9900
**Absorbtion Coeff. HTM**	$1/cm.eV^{1/2}$	3.7570	3.0613	3.1000	3.7569	2.9416	3.1000	3.7569
**CB DOS HTM**	$1/cm^3$ (10^{value})	19.3421	19.3560	18.0000	19.3420	19.4116	18.0000	19.3420
**VB DOS HTM**	$1/cm^3$ (10^{value})	18.0000	18.6980	19.0000	18.0000	18.7610	19.0000	18.0000
**Affinity Energy HTM**	eV	2.0663	1.7932	2.5000	2.0663	1.7996	2.5000	2.0663
**Dielectric Perm. HTM**	-	20.0000	4.1241	20.0000	20.0000	4.5051	20.0000	20.0000

Table S3

Full space optimized parameters for CIGS

CIGS for non-toxic ETM		Optimization - Gradient based (fmin)	
**Variable**	**Unit**	**Without thickness of CIGS absorber**	**With thickness of CIGS absorber**
**Thickness ETM**	µm	0.010001	0.010124
**Donor Conc. ETM**	$1/cm^3$ (10^{value})	18.998	18.957
**Acceptor Conc. ETM**	$1/cm^3$ (10^{value})	1.4922	0.72606
**Energy Gap ETM**	eV	2.9982	3.2456
**Affinity Energy ETM**	eV	4.2035	4.2138
**Dielectric Perm. ETM**	-	14.857	7.3719
**Electron Mobility ETM**	$cm^2/V.s$	12.484	18.676
**Hole Mobility ETM**	$cm^2/V.s$	382.09	12.841
**VB DOS ETM**	$1/cm^3$ (10^{value})	18.362	18.824
**CB DOS ETM**	$1/cm^3$ (10^{value})	18.001	18.763
**Absorbtion Coeff. ETM**	$1/cm.eV^{1/2}$	1001.5	18381
**Front Contact Work function**	eV	4.2035	4.2138
**Thickness CIGS Absorber**	µm		3.9999
